# Age-dependent prevalence of malocclusions requiring treatment according to the KIG classification: A multipart cross-sectional study over a 10-year period from the district of Viersen/North Rhine

**DOI:** 10.1007/s00056-024-00550-1

**Published:** 2024-10-02

**Authors:** Gero Stefan Michael Kinzinger, Jan Hourfar, Andrijana Maletic, Jörg Alexander Lisson

**Affiliations:** 1 Practice Essen, Essen, Germany; 2 Practice Michelstadt, Michelstadt, Germany; 3 Practice Goch, Goch, Germany; 4 International Medical College, University Duisburg-Essen, Duisburg-Essen, Germany; 5 Department of Orthodontics, Saarland University, 66424 Homburg/Saar, Germany

**Keywords:** Treatment timing, Index of orthodontic treatment need, KIG grade, DMS 6, Severity, Behandlungszeitpunkt, Index des kieferorthopädischen Behandlungsbedarfs, KIG-Grad, DMS 6, Schweregrad

## Abstract

**Background and aim:**

Patients with statutory health insurance (SHI) in Germany must undergo an assessment of orthodontic treatment need using the “Kieferorthopädische Indikationsgruppen” (KIG; orthodontic indication groups) classification system since 2002. A treatment need only exists if anomalies of a certain degree of severity are present. The aim of this study was to evaluate the age-dependent prevalence and percentage distribution of KIG grades requiring treatment in patients with SHI before the age of 18 over a 10-year period.

**Patients and methods:**

Between 2012 and 2021, treatment indication existed for 1951 (1025 female, 926 male) out of 2288 patients with SHI in the cohort of this study before the age of 18 according to current SHI guidelines. The KIG classification was based on the highest existing KIG grade. There were no multiple classifications. The patient cohort was divided into three patient groups (PG) according to chronological age for analysis: PG 1 < 10 years of age (early treatment), PG 2 10 to < 13 years of age (main treatment) and PG 3 13 to < 18 years of age (late treatment).

**Results:**

In PG 1 (454 patients), the KIG classifications D (26.5%), K (25.5%), M (19.4%), and P (18.0%) dominated. In PG 2 (998 patients), classifications D (33.2%), predominated, whereas K (7.5%) and M (5.9%) rarely occurred. The classifications E (12.6%) and P (13.3%) appeared quite frequently. Transverse deviations occurred only about half as often in PG 2 as in PG 1 and PG 3. In PG 3 (499 patients), the classification E (17.6%) was particularly common, while P (2.6%) was rare. The proportion of KIG grades 5 decreased depending on age: 19% in PG 1, 13.5% in PG 2, 10.4% in PG 3. The prevalence of sagittal classifications was highest in all age groups (45.9% in PG 1, 39.1% in PG 2, 31.5% in PG 3).

**Conclusions:**

The distribution of KIG classifications requiring treatment was not homogeneous, but age dependent. The differences were particularly evident in the early treatment group and may be due to the limited applicability of the KIG classification system in patients before late mixed dentition. With increasing age at initial examination, the prevalence of sagittal classifications decreased, while that of vertical classifications increased. Still, the sagittal classifications D and M occurred most frequently in all age groups. The KIG classification D was always the most common in all patients until the age of 18.

## Abstract (de)

**Hintergrund und Ziel:**

Seit dem 01. Januar 2002 wird in Deutschland bei der kieferorthopädischen Erstuntersuchung der Anspruch auf kieferorthopädische Leistungen bei gesetzlich versicherten Patienten durch Anwendung eines Indexsystems (Kieferorthopädische Indikationsgruppen, KIG) festgelegt. Dieser besteht nur, wenn Anomalien eines bestimmten Ausprägungsgrades vorliegen. Ziel dieser Untersuchung war die Evaluation altersabhängiger Prävalenzen und prozentualer Verteilung behandlungsbedürftiger KIG-Grade bei gesetzlich versicherten Patienten über einen Zeitraum von 10 Jahren.

**Patienten und Methoden:**

Zwischen 2012 und 2021 war bei 1951 (1025 weiblich, 926 männlich) von 2288 gesetzlich Versicherten in der Patientenkohorte dieser Studie vor Erreichen des 18. Lebensjahres nach aktuellen Richtlinien der GKV eine kieferorthopädische Behandlung angezeigt. Die KIG-Klassifikation fand in den höchsten bestehenden KIG-Grad statt. Mehrfacheinstufungen erfolgten nicht. Nach chronologischem Alter wurde das Kollektiv zur Analyse in 3 Patientengruppen (PG) unterteilt: PG 1 < 10. Lebensjahre (Frühbehandlung), PG 2 10. bis < 13. Lebensjahre (Hauptbehandlung) und PG 3 13. bis < 18. Lebensjahre (Spätbehandlung).

**Ergebnisse:**

In PG 1 (454 Patienten) dominierten die KIG-Indikationsgruppen D (26,5%), K (25,5%), M (19,4%) und P (18,0%). In PG 2 (998 Patienten) überwog die Indikationsgruppe D (33,2%), wogegen K (7,5%) und M (5,9%) selten befundet wurden. Die Klassifikationen E (12,6%) und P (13,3%) erschienen vergleichsweise häufig. Transversale Abweichungen traten in PG 2 nur etwa halb so oft auf wie in PG 1 und PG 3. In PG 3 (499 Patienten) war die Einstufung E (17,6%) besonders häufig, P (2,6%) dagegen selten. Der Anteil von KIG-Graden 5 nahm altersabhängig ab: 19% bei PG 1, 13,5% bei PG 2, 10,4% bei PG 3. Die Prävalenz sagittaler Klassifikationen war bei allen Altersgruppen am größten (45,9% in PG 1, 39,1% in PG 2, 31,5% in PG 3).

**Schlussfolgerungen:**

Die Verteilung behandlungsbedürftiger KIG-Klassifikationen bei unterschiedlichen Altersgruppen war nicht homogen, sondern altersabhängig. Die Unterschiede traten besonders in der Frühbehandlungsgruppe hervor und dürften auf die eingeschränkte Anwendbarkeit der KIG-Klassifikation bei Patienten vor der zweiten Wechselgebissphase zurückzuführen sein. Mit zunehmendem Alter bei Erstuntersuchung nahm die Prävalenz der sagittalen Klassifikationen ab, die der vertikalen Klassifikationen dagegen zu. Dennoch traten über alle Altersgruppen die sagittalen Klassifikationen D und M kombiniert am häufigsten auf. Die KIG-Klassifikation D kam bei allen Patienten bis zur Vollendung des 18. Lebensjahres stets am häufigsten vor.

## Introduction

The paragraph § 29.1 of SGB V (“Sozialgesetzbuch” [SGB] “fünf” [V]) sets the legal framework and regulations for orthodontic treatment of statutorily health insured (SHI) patients in Germany. Patients with SHI are entitled to orthodontic treatment if “there is a misalignment of the jaw or teeth that significantly impairs or threatens to impair chewing, biting, speaking or breathing” [1]. This very comprehensive entitlement has been restricted since 01 January 2002, by the introduction of the KIG classification system (“Kieferorthopädische Indikationsgruppen” [KIG], orthodontic indication groups [2]; Table 1). The KIG classifications are based on the index of orthodontic treatment need (IOTN index [3]). Five degrees of treatment need are to be delineated in the classification groups based on the clinical findings, whereby only KIG degrees 3–5 are entitled to treatment at the expense of the SHI. However, this is an exclusively cost-reducing exclusion. The medical necessity of treatment for KIG grades 1 and 2 is generally not questioned. Orthodontic measures before the start of the late mixed dentition and from the age of 18 onwards are only covered for a limited number of classifications and grades.

**Table 1:** “Kieferorthopädische Indikationsgruppen” (KIG; orthodontic indication groups) according to the guidelines of the Federal Committee of Dentists and Health Insurance Funds for orthodontic treatment (numbers in mm) Kieferorthopädische Indikationsgruppen (KIG) gemäß den Richtlinien des Bundesausschusses der Zahnärzte und Krankenkassen für die kieferorthopädische Behandlung (Zahlenangaben in mm)

KIG classification	Description	Grade 1	Grade 2	Grade 3	Grade 4	Grade 5
A	Craniofacial anomalies	–	–	–	–	(Cleft palate and syndromes)
U	Missing teeth (agenesis or loss)	–	–	–	Missing teeth	–
S	Eruption disorders	–	–	–	Impaction (except for third molars)	Displacement (except for third molars)
D	Sagittal discrepancy Increased overjet	≤ 3	3 <x ≤ 6	–	6 < x ≤ 9	> 9
M	Sagittal discrepancy Negative overjet	–	–	–	0 < x ≤ 3	> 3
O	Vertical discrepancy Open bite	≤ 1	1 < x ≤ 2	2 < x ≤ 4	> 4 Habitually open	> 4 Skeletally open
T	Vertical discrepancy Deep bite	1 < x ≤ 3	> 3 With/without mucosal contact	> 3 With traumatic mucosal impingement	–	–
B	Transverse discrepancy Scissors bite	–	–	–	Scissors bite	–
K	Transverse discrepancy Crossbite	–	Buccolingually cusp-to-cusp relation	Bilateral crossbite	Unilateral crossbite	–
E	Contact point displacement	< 1	1 < x ≤ 3	3 < x ≤ 5	> 5	–
P	Space deficiency	–	≤ 3	3 < x ≤ 4	> 4	–

Recent long-term studies have made it possible for the first time to draw detailed conclusions about the local frequency of individual groups of KIG classifications and degrees and their percentage at the time of the initial orthodontic examination [4–6]. Currently, there are only two single-session, differently designed clinical cross-sectional studies investigating preselected age groups in Germany that have evaluated the prevalence of malocclusions requiring treatment in accordance with valid SHI guidelines [7].

Glasl et al. [8] examined the prevalence and development of KIG classifications in 1251 schoolchildren (50.5% male, 49.5% female) aged between 9 and 11 years in Frankfurt/Main in 2004. They identified a treatment indication as defined by the SHI (KIG grades 3–5) in 41.4% of all cases. In this regional study, care was taken to ensure that the children had participated in the preliminary study by Schopf [9] from 2000 wherever possible.

As part of the Sixth German Oral Health Study (DMS 6) [10, 11], a survey of the prevalence of malocclusion in 8–9 year olds was conducted in 2021 in 16 study centers among 705 participants (51.4% male, 48.6% female) [12]. The proportion of 8 year olds was 49.4%, and 50.6% of 9 year olds. The proportion for whom orthodontic treatment was indicated (KIG grades ≥ 3) was 40.4% in DMS 6.

A long-term study with age-dependent subdivision of the patient cohort into different groups according to age at treatment begin for underage patients is not yet available.

## Objectives

The aims of this study were the following:

- Determine the prevalence and percentage distribution of KIG classifications and grades requiring treatment in an orthodontic practice in North Rhine/Germany in statutorily insured patients aged < 18 years over a period of 10 years,
- Determine whether certain prevalences of KIG classifications and grades were age dependent, and
- Scrutinize the prevalence and percentage distribution depending on the periods of early, regular, and late treatment.

## Patients and methods

A total of 2288 patients with statutory health insurance (SHI) presented at an orthodontic specialist practice in the district of Viersen/North Rhine for consultation and KIG classification between 2012 and 2021. In the presence of KIG grades 3–5, orthodontic treatment was indicated before the age of 18 according to the current guidelines of the SHI.

Depending on chronological age, the patient collective was divided into three groups analogous to the treatment period:

- PG 1: < 10 years of age (early treatment),
- PG 2: 10 to < 13 years of age (main treatment), and
- PG 3: 13 to < 18 years of age (late treatment).

### Classification of orthodontic treatment need using KIG

Possible tooth and jaw malposition are subdivided into 11 classifications of the KIG system. Each classification is additionally subdivided into five grades. Since only grades 3–5 are eligible for treatment, 19 possible combinations of classification and grade trigger cost reimbursement through the SHI. The ranking starts with A as the highest and P as the lowest possible classification (Table 1). The classifications D + M (sagittal), O + T (vertical), and B + K (transverse) can be combined according to spatial planes, as well as E + P as dental malpositions.

The diagnoses were solely recorded through clinical inspection, as required by legislation. The extent and direction of sagittal and vertical overjet, anterior crowding, and space deficits were measured intraorally using sliding calipers Münchner Modell® (Dentaurum, Ispringen, Germany) with a precision of 0.25 mm. The assessment of occlusion regarding frontal and lateral crossbites was performed visually. Only if justified by clinical reasons were x‑rays made to diagnose possible aplasia, retention, or displacement of permanent teeth.

Children and adolescents up to the age of 18 were examined. The classification of the patients into the respective KIG grades 3–5 with treatment need according to the valid SHI guidelines [1] always took place in the highest of the 19 possible variants. There were no multiple responses in the present study. Exclusively two orthodontists recorded the KIG classifications and grades during the entire observation period, applying the four-eye principle.

### Statistics

Anonymized patient data were collected using a spreadsheet software (Excel®, Microsoft Corp., Redmond, WA, USA). Normal distribution of the variable “age” was evaluated graphically and using the Shapiro–Wilk test with SPSS® (version 28 for Windows®; IBM Corp., Armonk, NY, USA). Mean and standard deviation were recorded. All other data were interpreted descriptively.

## Results

In all, 1951 (85.3%) of 2288 patients before the age of 18 were eligible for orthodontic treatment according to the applicable guidelines. The average age of the 1025 (52.5%) female and 926 (47.5%) male patients was 11.59 ± 2.28 years (min. 3 years 6 months/max. 17 years 11 months). The age distribution shows a peak between the ages of 10 and 12 over the entire period (Table 2).

**Table 2:** Age and gender distribution of 1951 statutorily insured patients between 2012 and 2021 with initial orthodontic consultation before the age of 18, and with “Kieferorthopädische Indikationsgruppen” (KIG) grades 3, 4, and 5 Alters- und Geschlechterverteilung der 1951 zwischen 2012 und 2021 in die Studie inkludierten gesetzlich versicherten kieferorthopädischen Erstberatungspatienten vor Vollendung des 18. Lebensjahres mit KIG(kieferorthopädische Indikationsgruppen)-Graden 3, 4 und 5

Gender distribution [*n*]				Mean patient age [years]	Patient distribution according to age (years) [*n*]											
Observed period	Female	Male	Total	M ± SD	≤ 6	7	8	9	10	11	12	13	14	15	16	17
2012–2021	1025	926	1951	11.59 ± 2.28	29	87	135	203	337	349	312	222	131	77	34	35

*M* Mean, *SD* standard deviation

The patients could be distributed into the previously defined groups as follows:

- PG 1: 454 patients (256 female, 198 male, min. 3 years 6 months, max. 9 years 11 months),
- PG 2: 998 patients (546 female, 452 male, min. 10 years 0 months, max. 12 years 11 months), and
- PG 3: 499 patients (223 female, 276 male, min. 13 years 0 months, max. 17 years 11 months).

The number of patients in the groups early and late treatment together corresponded approximately to the number of patients in the group main treatment.

### All patients

#### Frequency of KIG classifications (Fig. 1a; Table 3)

In the 10-year period, 554 (28.4%) patients had the KIG classification D. The KIG classifications K (*n* = 262, 13.4%), P (*n* = 228, 11.7%), E (*n* = 222, 11.4%), S (*n* = 217, 11.1%), and M (*n* = 201, 10.3%) accounted for more than 10%.

**Fig. 1:**
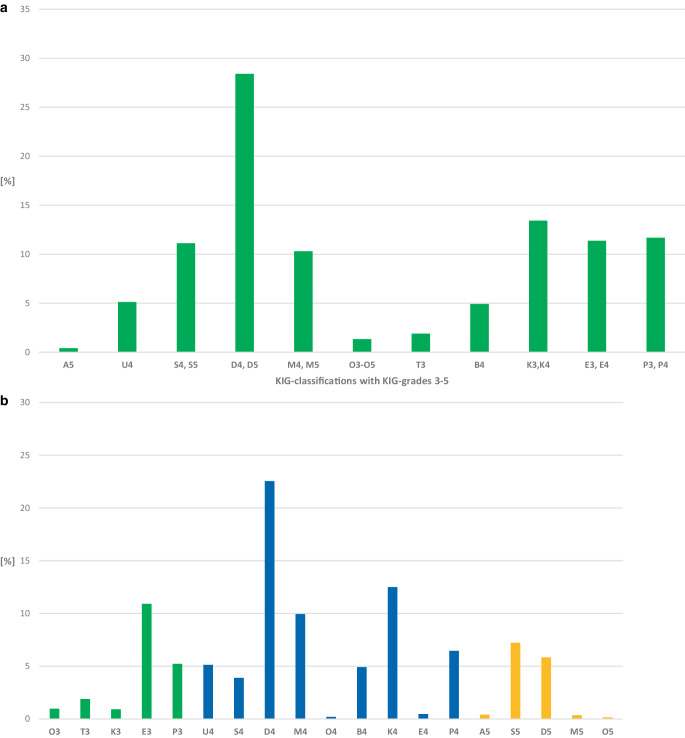
**a** Percentages of the 11 “Kieferorthopädische Indikationsgruppen” (KIG) classifications among all statutorily insured patients until the age of 18 between 2012 and 2021. **b** Percentages of the 19 KIG grades 3–5 among all statutorily insured patients until the age of 18 between 2012 and 2021. See Table 3 for description of classifications **a** Prozentuale Häufigkeit der 11 verschiedenen kieferorthopädischen Indikationsgruppen (KIG) bei allen gesetzlich versicherten Patienten vor Vollendung des 18. Lebensjahres im Zeitraum 2012–2021. **b** Prozentuale Häufigkeit der 19 verschiedenen KIG-Grade 3–5 bei allen gesetzlich versicherten Patienten vor Vollendung des 18. Lebensjahres im Zeitraum 2012–2021. Beschreibung der Klassifikationen in Tab. 3

**Table 3:** Frequency and percentage of the “Kieferorthopädische Indikationsgruppen” (KIG) classification with treatment eligibility (11 classification and 19 grades) in all statutorily insured patients before the age of 18 between 2012 and 2021 Häufigkeit und prozentuale Verteilung der verschiedenen behandlungsbedürftigen KIG(kieferorthopädische Indikationsgruppen)-Befunde (11 Indikationsgruppen und 19 Behandlungsbedarfsgrade) bei allen Patienten vor Vollendung des 18. Lebensjahres im Zeitraum 2012–2021

KIG classification	Description	Grade 3		Grade 4		Grade 5		Grades 3–5	
		[*n*]	[%]	[*n*]	[%]	[*n*]	[%]	[*n*]	[%]
A	Craniofacial anomalies	–	–	–	–	8	0.4	8	0.4
U	Missing teeth (agenesis or loss)	–	–	100	5.1	–	–	100	5.1
S	Eruption disorders	–	–	76	3.9	141	7.2	217	11.1
D	Sagittal discrepancy Increased overjet	–	–	440	22.6	114	5.8	554	28.4
M	Sagittal discrepancy Negative overjet	–	–	194	9.9	7	0.4	201	10.3
O	Vertical discrepancy Open bite	19	1.0	4	0.2	3	0.2	26	1.4
T	Vertical discrepancy Deep bite	37	1.9	–	–	–	–	37	1.9
B	Transverse discrepancy Scissors bite	–	–	96	4.9	–	–	96	4.9
K	Transverse discrepancy Crossbite	18	0.9	244	12.5	–	–	262	13.4
E	Contact point displacement	213	10.9	9	0.5	–	–	222	11.4
P	Space deficiency	102	5.2	126	6.5	–	–	228	11.7
Total	–	389	19.9	1289	66.1	273	14.0	1951	100.0

The KIG classification U (*n* = 100, 5.1%) accounted for more than 5%, B (*n* = 96, 4.9%), T (*n* = 37, 1.9%), and O (*n* = 26, 1.4%) for more than 1%, and A (*n* = 8, 0.4%) for less than 1%.

Of 11 possible KIG classifications, 86.3% were distributed among the 6 most frequent (D, K, P, E, S, and M) and 13.7% among the 5 least frequent (U, B, T, O, and A).

#### Frequency of KIG grades 3–5 (Fig. 1b; Table 3)

Of the 19 possible KIG grades that triggered treatment, D4 occurred most frequently (*n* = 440, 22.6%). The KIG grades K4 (*n* = 244, 12.5%) and E3 (*n* = 213, 10.9%) accounted for more than 10%, and S5 (*n* = 141, 7.2%), P4 (*n* = 126, 6.5%), D5 (*n* = 114, 5.8%), P3 (*n* = 102, 5.2%), and U4 (*n* = 100, 5.1%) for more than 5%.

The proportion of the 8 most common KIG grades together was, therefore, 75.8%.

Of 1951 patients, 19.9% had pronounced malocclusions (KIG grade 3), 66.1% had very pronounced malocclusions (KIG grade 4) and 14.0% had extremely pronounced malocclusions (KIG grade 5).

#### Classification according to spatial planes and tooth malposition (Table 7)

The sagittal classifications D and M accounted for 38.7%, the vertical O and T for 3.3%, and the transverse B and K for 18.3% of all anomalies requiring treatment. The classifications E and P together reached a frequency of 23.1%.

### PG 1

#### Frequency of KIG classifications (Fig. 2a; Table 4)

In the 10-year period, 120 (26.5%) of 454 patients in PG 1 had the KIG classification D and 116 (25.5%) had the KIG classification K. Proportionally more than 10% were allocated to the KIG classifications M (*n* = 88, 19.4%) and P (*n* = 82, 18.0%), proportionally more than 1% to S (*n* = 13, 2.9%), U (*n* = 9, 2.0%), E (*n* = 9, 2.0%), O (*n* = 6, 1.3%), and A (*n* = 6, 1.3%), less than 1% to B (*n* = 4, 0.9%) and T (*n* = 1, 0.2%).

**Fig. 2:**
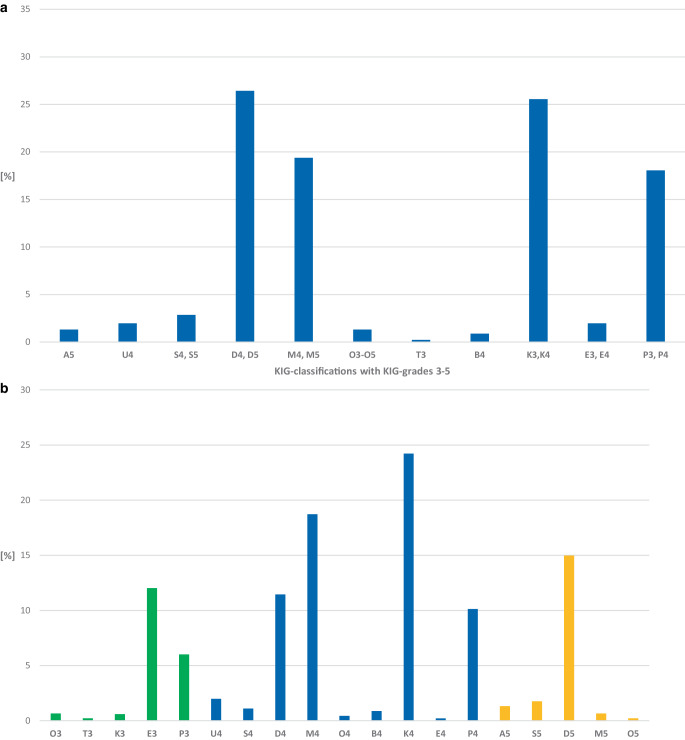
**a** Percentages of the 11 “Kieferorthopädische Indikationsgruppen” (KIG) classifications among statutorily insured patients of group PG 1 until the age of 18 between 2012–2021. **b** Percentages of the 19 KIG grades 3–5 among statutorily insured patients of group PG 1 until the age of 18 between 2012–2021. See Table 4 for description of classifications **a** Prozentuale Häufigkeit der 11 verschiedenen KIG(kieferorthopädische Indikationsgruppen)-Befunde bei gesetzlich versicherten Patienten der Gruppe PG 1 im Zeitraum 2012–2021. **b** Prozentuale Häufigkeit der 19 verschiedenen KIG-Grade 3–5 bei gesetzlich versicherten Patienten der Gruppe PG 1 im Zeitraum 2012–2021. Beschreibung der Klassifikationen in Tab. 4

**Table 4:** Frequency and percentage of the “Kieferorthopädische Indikationsgruppen” (KIG) classification with treatment eligibility (11 classification and 19 grades) in patient group (PG) 1 between 2012 and 2021 Häufigkeit und prozentuale Verteilung der verschiedenen behandlungsbedürftigen KIG(kieferorthopädische Indikationsgruppen)-Befunde (11 Indikationsgruppen und 19 Behandlungsbedarfsgrade) bei Patienten der Gruppe PG 1 im Zeitraum 2012–2021

KIG classification	Description	Grade 3		Grade 4		Grade 5		Grades 3–5	
		[*n*]	[%]	[*n*]	[%]	[*n*]	[%]	[*n*]	[%]
A	Craniofacial anomalies	–	–	–	–	6	1.3	6	1.3
U	Missing teeth (agenesis or loss)	–	–	9	2.0	–	–	9	2.0
S	Eruption disorders	–	–	5	1.1	8	1.8	13	2.9
D	Sagittal discrepancy Increased overjet	–	–	52	11.5	68	15.0	120	26.5
M	Sagittal discrepancy Negative overjet	–	–	85	18.7	3	0.7	88	19.4
O	Vertical discrepancy Open bite	3	0.7	2	0.4	1	0.2	6	1.3
T	Vertical discrepancy Deep bite	1	0.2	–	–	–	–	1	0.2
B	Transverse discrepancy Scissors bite	–	–	4	0.9	–	–	4	0.9
K	Transverse discrepancy Crossbite	6	1.3	110	24.2	–	–	116	25.5
E	Contact point displacement	8	1.8	1	0.2	–	–	9	2.0
P	Space deficiency	36	7.9	46	10.1	–	–	82	18.0
Total	–	54	11.9	314	69.1	86	19.0	454	100.0

Of 11 possible KIG classifications, 52% were distributed among the 2 most frequent (D and K), 89.4% among the 4 most frequent (D, K, M, and P), and 10.6% among the 7 least frequent (S, U, E, O, A, B, and T).

#### Frequency of KIG grades 3–5 (Fig. 2b; Table 4)

K4 occurred most frequently with 24.2% (*n* = 110). M4 (*n* = 85, 18.7%), D5 (*n* = 68, 15.0%), D4 (*n* = 52, 11.5%), and P4 (*n* = 46, 10.1%) accounted for more than 10%, and P3 (*n* = 36, 7.9%) for more than 5%. The combined share of the 6 most frequent treatment requirement grades was 87.3%.

Of 454 patients from PG 1, 11.9% had severe malocclusions (KIG grade 3), 69.1% had severe malocclusions (KIG grade 4), and 19.0% had extremely severe malocclusions (KIG grade 5).

#### Subdivision according to spatial planes and tooth malposition (Table 7)

The sagittal classifications D and M accounted for 45.9%, the vertical O and T for 1.5%, and the transverse B and K for 26.4% of all anomalies requiring treatment. The classifications E and P together reached a frequency of 20.0%.

### PG 2

#### Frequency of KIG classifications (Fig. 3a; Table 5)

In the 10-year period, 331 (33.2%) of 998 patients in PG 2 had the KIG classification D. Proportionally more than 10% were allocated to the KIG classifications S (*n* = 141, 14.1%), P (*n* = 133, 13.3%), and E (*n* = 125, 12.6%), proportionally more than 5% to K (*n* = 75, 7.5%), M (*n* = 59, 5.9%), U (*n* = 57, 5.7%), proportionally more than 1% to B (*n* = 43, 4.3%), T (*n* = 21, 2.1%), and O (*n* = 12, 1.2%), less than 1% to A (*n* = 1, 0.1%).

**Fig. 3:**
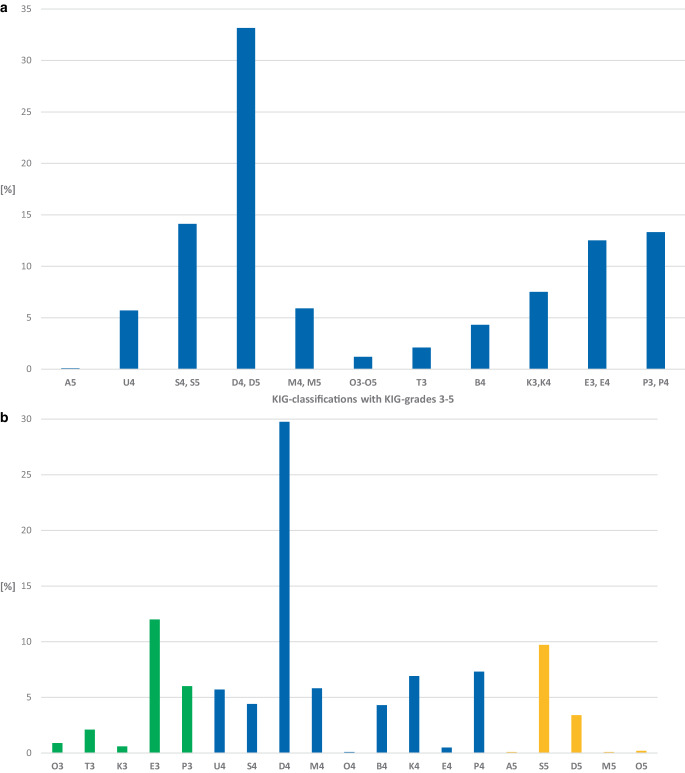
**a** Percentages of the 11 “Kieferorthopädische Indikationsgruppen” (KIG) classifications among statutorily insured patients of group 2 (PG 2) until the age of 18 between 2012 and 2021. **b** Percentages of the 19 KIG grades 3–5 among statutorily insured patients of group PG 2 until the age of 18 between 2012 and 2021. See Table 5 for description of classifications **a** Prozentuale Häufigkeit der 11 verschiedenen KIG(kieferorthopädische Indikationsgruppen)-Befunde bei gesetzlich versicherten Patienten der Gruppe PG 2 im Zeitraum 2012–2021. **b** Prozentuale Häufigkeit der 19 verschiedenen KIG-Grade 3–5 bei gesetzlich versicherten Patienten der Gruppe PG 2 im Zeitraum 2012–2021. Beschreibung der Klassifikationen in Tab. 5

**Table 5:** Frequency and percentage of the “Kieferorthopädische Indikationsgruppen” (KIG) classification with treatment eligibility (11 classification and 19 grades) in patient group 2 (PG 2) between 2012 and 2021 Häufigkeit und prozentuale Verteilung der verschiedenen behandlungsbedürftigen KIG(kieferorthopädische Indikationsgruppen)-Befunde (11 Indikationsgruppen und 19 Behandlungsbedarfsgrade) bei Patienten der Gruppe PG 2 im Zeitraum 2012–2021

KIG classification	Description	Grade 3		Grade 4		Grade 5		Grades 3–5	
		[*n*]	[%]	[*n*]	[%]	[*n*]	[%]	[*n*]	[%]
A	Craniofacial anomalies	–	–	–	–	1	0.1	1	0.1
U	Missing teeth (agenesis or loss)	–	–	57	5.7	–	–	57	5.7
S	Eruption disorders	–	–	44	4.4	97	9.7	141	14.1
D	Sagittal discrepancy Increased overjet	–	–	297	29.8	34	3.4	331	33.2
M	Sagittal discrepancy Negative overjet	–	–	58	5.8	1	0.1	59	5.9
O	Vertical discrepancy Open bite	9	0.9	1	0.1	2	0.2	12	1.2
T	Vertical discrepancy Deep bite	21	2.1	–	–	–	–	21	2.1
B	Transverse discrepancy Scissors bite	–	–	43	4.3	–	–	43	4.3
K	Transverse discrepancy Crossbite	6	0.6	69	6.9	–	–	75	7.5
E	Contact point displacement	120	12.1	5	0.5	–	–	125	12.6
P	Space deficiency	60	6.0	73	7.3	–	–	133	13.3
Total	–	216	21.7	647	64.8	135	13.5	998	100.0

Of 11 possible KIG classifications, 73.2% were distributed among the 4 most frequent (D, S, P, and E) and 26.8% among the 7 least frequent (K, M, U, B, T, O, and A).

#### Frequency of KIG grades 3–5 (Fig. 3b; Table 5)

D4 occurred most frequently with 29.8% (*n* = 297). More than 10% were distributed among E3 (*n* = 120, 12.1%), more than 5% among S5 (*n* = 97, 9.7%), P4 (*n* = 73, 7.3%), K4 (*n* = 69, 6.9%), P3 (*n* = 60, 6.0%), M4 (*n* = 58, 5.8%), and U4 (*n* = 57, 5.7%).

The combined share of the 8 most frequent treatment requirement grades was 83.3%.

Of 998 patients from PG 2, 21.7% had pronounced malocclusions (KIG grade 3), 64.8% had very pronounced malocclusions (KIG grade 4) and 13.5% had extremely pronounced malocclusions (KIG grade 5).

#### Subdivision according to spatial planes and tooth malposition (Table 7)

The sagittal classifications D and M represented 39.1%, the vertical O and T 3.3% and the transverse B and K 11.8% of all anomalies requiring treatment. The classifications E and P together reached a frequency of (25.9%).

### PG 3

#### Frequency of KIG classifications (Fig. 4a; Table 6)

In the 10-year period, 103 (20.6%) of 499 patients in PG 3 had the KIG classification D. The KIG classifications E (*n* = 88, 17.6%), K (*n* = 71, 14.3%), S (*n* = 63, 12.6%) and M (*n* = 54, 10.9%) accounted for more than 10%. More than 5% were represented by B (*n* = 49, 9.8%) and U (*n* = 34, 6.8%), more than 1% by T (*n* = 15, 3.0%), P (*n* = 13, 2.6%), and O (*n* = 8, 1.6%), less than 1% by A (*n* = 1, 0.2%).

**Fig. 4:**
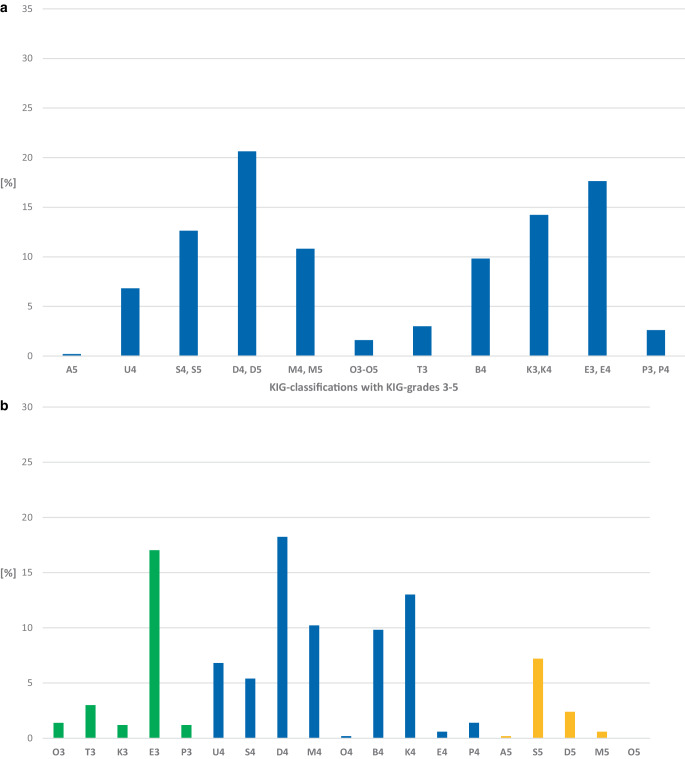
**a** Percentages of the 11 “Kieferorthopädische Indikationsgruppen” (KIG) classifications among statutorily insured patients of group 3 (PG 3) until the age of 18 between 2012 and 2021. **b** Percentages of the 19 KIG grades 3–5 among statutorily insured patients of group PG 3 until the age of 18 between 2012 and 2021. See Table 6 for description of classifications **a** Prozentuale Häufigkeit der 11 verschiedenen KIG(kieferorthopädische Indikationsgruppen)-Befunde bei gesetzlich versicherten Patienten der Gruppe PG 3 im Zeitraum 2012–2021. **b** Prozentuale Häufigkeit der 19 verschiedenen KIG-Grade 3–5 bei gesetzlich versicherten Patienten der Gruppe PG 3 im Zeitraum 2012–2021. Beschreibung der Klassifikationen in Tab. 6

**Table 6:** Frequency and percentage of the “Kieferorthopädische Indikationsgruppen” (KIG) classification with treatment eligibility (11 classification and 19 grades) in patient group 3 (PG 3) between 2012 and 2021 Häufigkeit und prozentuale Verteilung der behandlungsbedürftigen KIG(kieferorthopädische Indikationsgruppen)-Befunde (11 Indikationsgruppen und 19 Behandlungsbedarfsgrade) bei Patienten der Gruppe PG 3 im Zeitraum 2012–2021

KIG classification	Description	Grade 3		Grade 4		Grade 5		Grades 3–5	
		[*n*]	[%]	[*n*]	[%]	[*n*]	[%]	[*n*]	[%]
A	Craniofacial anomalies	–	–	–	–	1	0.2	1	0.2
U	Missing teeth (agenesis or loss)	–	–	34	6.8	–	–	34	6.8
S	Eruption disorders	–	–	27	5.4	36	7.2	63	12.6
D	Sagittal discrepancy Increased overjet	–	–	91	18.2	12	2.4	103	20.6
M	Sagittal discrepancy Negative overjet	–	–	51	10.3	3	0.6	54	10.9
O	Vertical discrepancy Open bite	7	1.4	1	0.2	0	0.0	8	1.6
T	Vertical discrepancy Deep bite	15	3.0	–	–	–	–	15	3.0
B	Transverse discrepancy Scissors bite	–	–	49	9.8	–	–	49	9.8
K	Transverse discrepancy Crossbite	6	1.2	65	13.1	–	–	71	14.3
E	Contact point displacement	85	17.0	3	0.6	–	–	88	17.6
P	Space deficiency	6	1.2	7	1.4	–	–	13	2.6
Total	–	119	23.8	328	65.8	52	10.4	499	100.0

Of 11 possible KIG classifications, 76.0% were distributed among the 5 most frequent (D, E, K, S, and M) and 24.0% among the 6 least frequent (B, U, T, P, O, and A).

#### Frequency of KIG grades 3–5 (Fig. 4b; Table 6)

Proportionally more than 10% were distributed to D4 (*n* = 91, 18.2%), E3 (*n* = 85, 17.0%), K4 (*n* = 65, 13.1%), and M4 (*n* = 51, 10.3%); more than 5% to B4 (*n* = 49, 9.8%), S5 (*n* = 36, 7.2%), U4 (*n* = 34, 6.8%), and S4 (*n* = 27, 5.4%).

The combined proportion of the 8 most frequent treatment requirement grades was 87.8%.

Of 499 patients from PG 3, 23.8% had pronounced malocclusions (KIG grade 3), 65.8% had very pronounced malocclusions (KIG grade 4), and 10.4% had extremely pronounced malocclusions (KIG grade 5).

#### Subdivision according to spatial planes and tooth malposition (Table 7)

The sagittal classifications D and M represented 31.5%, the vertical O and T 4.6%, and the transverse B and K 24.1% of all anomalies requiring treatment. The classifications E and P together reached a frequency of 20.2%.

**Table 7:** Percentage distribution according to spatial planes and tooth position anomalies in all patients and by age-dependent subdivision between 2012 and 2021 Prozentuale Verteilung der Malokklusionen nach Raumebenen bzw. Zahnstellungsanomalien bei allen Patienten und nach altersabhängiger Unterteilung im Zeitraum 2012–2021

Combined KIG classifications^a^	All patients	All patients	< 10 years	< 10 years	10–< 13 years	10– < 13 years	13– < 18 years	13– < 18 years
	*n*	%	*n*	%	*n*	%	*n*	%
D + M (sagittal)	755	38.7	208	45.9	390	39.1	157	31.5
O + T (vertical)	63	3.3	7	1.5	33	3.3	23	4.6
B + K (transverse)	358	18.3	120	26.4	118	11.8	120	24.1
E + P (tooth malposition)	450	23.1	91	20.0	258	25.9	101	20.2
Total	1626	83.4	426	93.8	799	80.1	401	80.4

*KIG* “Kieferorthopädische Indikationsgruppen”

^a^See Table 3 for description of classifications

## Discussion

### Study limitations

The present study is not an epidemiological survey because the patients were predominantly referred for initial consultation and assessment by dentists over a 10-year period, but also by pediatricians and otolaryngology (ENT) specialists. Therefore, the preselected patient clientele is only representative to a limited extent. At 14.7%, the percentage of patients with KIG grades ≤ 2 was significantly lower than in the study by Glasl et al. [8] and in the DMS 6 [10].

A possible limitation of the methodology could be that the KIG classifications were set by two different examiners. According to Gesch et al. [13], there are considerable interexaminer differences in the classification of subjects into the respective indication groups and, thus, also different classifications into KIG grades < 3 and > 2 in borderline cases. Different data collection methods (clinic/dental cast) in the assessment of the malocclusion by different or orthodontically inexperienced examiners may have an unfavorable influence on examiner agreement. For this reason, KIG classifications were made according to the four-eye principle without exception. Especially in borderline cases, classifications were made based on a dental cast and, if necessary, a panoramic x‑ray.

### Results of the present study, total collective, intergroup comparison

The results of the total collective of 1951 patients during the scrutinized 10-year period correspond to those of previously performed studies by the same authors, both in terms of age distribution and the prevalence of KIG classifications and grades [4–6].

**Fig. 5:**
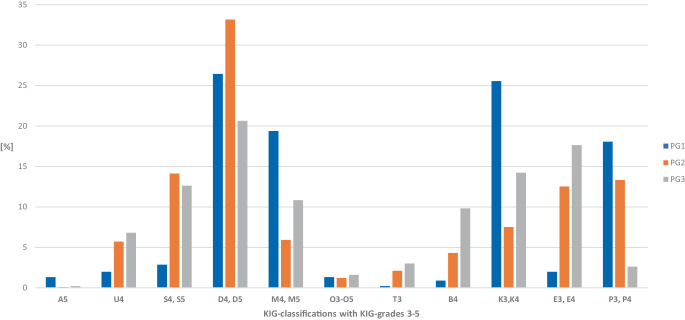
Percentages of the 11 “Kieferorthopädische Indikationsgruppen” (KIG) classifications among patients with statutory health insurance between 2012 and 2021: comparison of groups PG 1, PG 2, and PG 3 Prozentuale Anteile der 11 kieferorthopädischen Indikationsgruppen (KIG) bei gesetzlich versicherten Patienten im Zeitraum 2012–2021: direkter Vergleich der Gruppen PG 1, PG 2 und PG 3

**Fig. 6:**
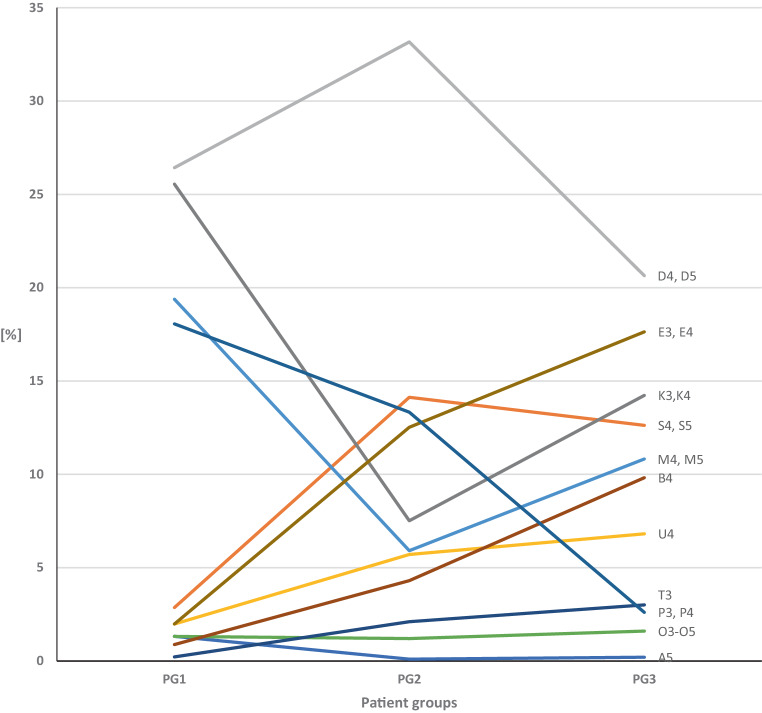
Comparison of percentages of 11 “Kieferorthopädische Indikationsgruppen” (KIG) classifications with grades 3–5 in groups PG 1, PG 2, and PG 3 Vergleich der prozentualen Anteile der 11 verschiedenen KIG (kieferorthopädische Indikationsgruppen)-Klassifikationen mit Graden 3–5 in den Gruppen PG 1, PG 2 und PG 3

However, when comparing the groups according to age at the start of treatment, differences became evident: The KIG classifications, K, M, and P were particularly common in the early treatment group PG 1, while T, B, U, E, and S were comparatively rare. In the 10–13 year olds (PG 2), the percentage of D was particularly high, while that of K and M was particularly low. In late treatments (PG 3), E and B occurred rather often, while P occurred particularly rarely (Figs. 5 and 6). Although D was the most frequent classification in all groups, its share was lower in patients treated late. This could be because the mandible grows the most of all facial bones postnatally and shows the greatest morphological changes [14], which may contribute to the reduction of the detectable overjet.

For 19 possible KIG grades 3–5, it was striking that only 6 out of those triggered treatment in 87.3% of patients in the early treatment group. Of these, 5 had a share of over 10%, with K4 being particularly high at 24.2%.

To achieve a comparable percentage (87.8%), 8 grades were required in the late treatment group, half of which were between 5 and 10% and half between 10 and 20%. In the main treatment group PG2, the KIG grade D4 dominated with 29.8%.

**Fig. 7:**
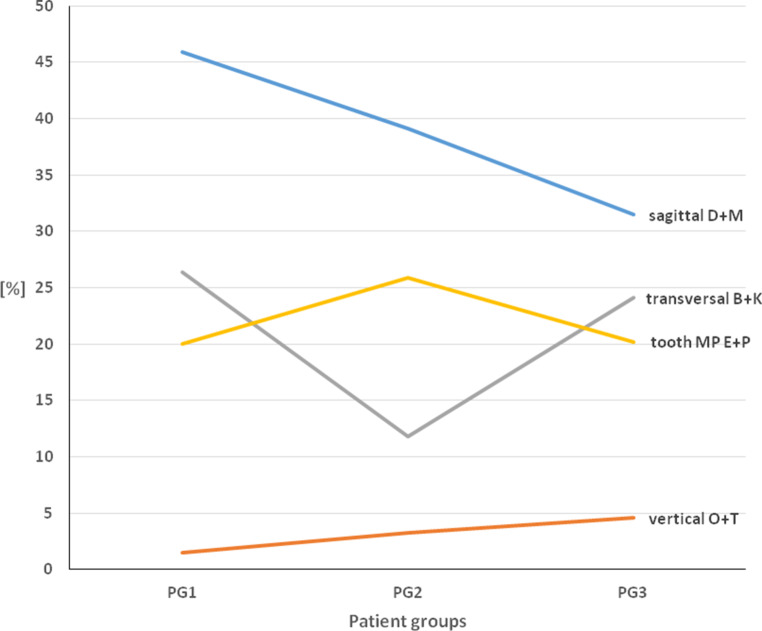
Comparison of percentages according to spatial planes and tooth malposition (sagittal D + M, vertical O + T, transverse B + K, tooth malposition E + P) in groups PG 1, PG 2, and PG 3. See Table 3 for description of classifications Vergleich der prozentualen Anteile der Malokklusionen nach Raumebenen bzw. Zahnstellungsanomalien (sagittal D + M, vertikal O + T, transversalB + K, Zahnfehlstellung E + P) in den Gruppen PG 1, PG 2 und PG 3. Beschreibung der Klassifikationen in Tab. 3

In the subdivision according to spatial planes and tooth position anomalies (Fig. 7), the prevalence of sagittal classifications according to D and M was greater than that of transverse classifications B and K, and vertical classifications O and T in all age groups. Classifications E and P occurred particularly frequently between the ages of 10 and 13 and, thus, in the main treatment. In contrast, transverse classifications B and K occurred only about half as frequently as in early and late treatment.

In that respect, however, the restrictions of the indication group system regarding treatment before reaching the late mixed dentition phase must be considered [1]. During this period, which in the present study mainly concerns PG 1 patients, only 8 KIG grades (D5, M4, M5, B4, K3, K4, P3, P4) trigger treatment according to SHI regulations. This leads to a lower incidence with a simultaneously higher occurrence of grades 4 and 5. These 8 KIG grades triggering treatment before the late mixed dentition phase had a correspondingly high proportion in PG 1 (78.8%), which was significantly lower in the other groups (PG 2: 34.7%, PG 3: 40.0%).

### Comparability of the methodology with existing studies

Differences are present when comparing the current results with previous cross-sectional studies during a limited timespan conducted in Germany [8, 10] to determine the need for treatment and the prevalence of malocclusions. It should be noted that other parameters were used as a basis and the study clientele was different. In those studies [8, 10], the age span of the examined patients was small, but they were not preselected. The tooth position and jaw anomalies were recorded exclusively clinically. Neither X‑ray documentation nor dental casts were available to confirm the diagnosis. Unlike in the present study, it was therefore not possible to record all available KIG classifications: classification A is missing in the study from Glasl et al. [8], because aplasia was not recorded, and retention or displacement of permanent teeth was only recorded indirectly. Classifications S and U are missing in the DMS 6 [10]. In contrast to the procedure in this study and as is usual in clinical practice, not only the highest degree of severity was recorded in the existing single-stage comparative studies [8, 10], but each possible KIG classification and each grade ≥ 3 were recorded separately, so that multiple responses of different severity levels were possible for individual study participants. This may lead to overrepresentation of certain anomalies. For patients in the early mixed dentition phase, the restricted KIG system for this dentition stage by the SHI regulations was not applied.

The DMS 6 was conducted in 16 study centers nationwide [12] to represent a national average. In contrast, the present study, like that of Glasl et al. [8], was regionally limited to the respective catchment area. However, current studies show that there are no regional peculiarities in KIG classifications—at least in the district of Viersen/North Rhine—and that both the prevalence and age distribution of KIG grades 3–5 requiring treatment correspond to the national average [5, 6].

### Comparability of the results with existing studies

The results of existing epidemiological studies [8, 10] are consistent with this study in that most patients requiring treatment show a KIG grade 4. Broken down by tooth position and jaw anomalies, the sagittal classifications D and M were most frequently represented in all age groups, as in the present study. In the vertical classifications, the KIG grade T3 was overrepresented both in the DMS 6 [10] and in the study by Glasl et al. [8]. One reason for this is likely to be the multiple responses to classifications. As a result, individual KIG classifications, such as here specifically an anomaly with KIG grade 3, may be overrepresented. If the recording procedure complies with the SHI rules and only one classification is made in the highest existing KIG grade, a classification in KIG grade 4 or 5 is likely to have been made much more frequently. This is particularly true for early treatment with its restricted indication system.

Rijpstra and Lisson [15] also discussed the fact that categorization into the KIG grade T3 is objectively very difficult and is, therefore, rarely carried out. In the index of orthodontic treatment need (IOTN), any tooth contact with the mucosa is already considered as T3; clearly visible impressions in the gingiva are considered T4 [3]. In Germany, 4.5 years after the introduction of the KIG classification system, certain wordings of the law were clarified [16]. Since then, impressions in the opposing jaw mucosa have not been considered an indication for treatment at the expense of the SHI funds. Only if this contact is traumatic and has led to recessions or other permanent damage to the periodontium is it considered classification T3. As problems with recessions and inflammation of the mucosa usually only occur with advancing age [17], it is not surprising that T3 was rarely ever found in long-term studies [5, 6] and in the present study.

It must be regarded that the KIG classification system was not primarily developed as an epidemiological index. Rather, it represents a control tool to determine individually whether treatment can be provided at the expense of the SHI for patients from the late mixed dentition onwards. The restriction to 8–9 year olds in the DMS 6 [10] and 9–11 year olds in the study by Glasl et al. [8] is, therefore, not uncritical, as malocclusions become more pronounced with increasing age during growth [18, 19]. Thus, in both studies with age-restricted examination clientele [8, 10], there is a risk of underestimating the actual prevalence of malocclusions and, thus, orthodontic treatment need.

To avoid this underestimation in this study, the patients were divided into three age groups. Here, however, different age-dependent changes could be seen: the sagittal classifications (especially D5) decreased in prevalence with age, while the vertical classifications (especially T3) increased. The transverse classifications (B and K) and the classifications relating to tooth position anomalies (E and P) showed a significantly different frequency in the age group between 10 and 13 years than in early and late treatment groups.

It remains, however, difficult to draw appropriate conclusions from findings regarding KIG classification and grade A5. Rijpstra and Lisson [15] found that malocclusions with KIG grade 5 were overrepresented in their study due to the frequent classification A5. The most frequent malformation in the KIG classification A5 was cleft lip, jaw, and palate. All pathologies leading to KIG A5 have extensive findings in the dentoalveolar area, which ought to be treated in an interdisciplinary manner, often at university settings with specialized treatment centers. This again must lead to an overrepresentation of KIG A5 in any study coming out of a university. However, the billing data of the Kassenzahnärztliche Bundesvereinigung [10] show a nationwide occurrence of only 0.3% of patients with KIG A5, so that the available figures correspond to the national average.

## Conclusion

The results allow the conclusion that the distribution of KIG classifications requiring treatment in patients before the age of 18 is not homogeneous but varies according to age. The differences were particularly evident in the early treatment group and were probably due to the limited applicability of the KIG classification in patients before the late mixed dentition phase. With increasing age at initial examination, the prevalence of sagittal classifications—here especially D5—decreased, while vertical classifications—here especially T3—increased. Nevertheless, the sagittal classifications D and M together were the most common across all age groups. The KIG classification D always occurred most frequently in patients before the age of 18.
